# Genetic and epigenetic profiling identifies two distinct classes of spinal meningiomas

**DOI:** 10.1007/s00401-022-02504-6

**Published:** 2022-09-27

**Authors:** Franz L. Ricklefs, Krystian D. Fita, Malte Mohme, Christian Mawrin, Ramin Rahmanzade, Felix Sahm, Lasse Dührsen, Carolin Göbel, Katrin Lamszus, Manfred Westphal, Ulrich Schüller, Sven O. Eicker

**Affiliations:** 1 Department of Neurosurgery, University Medical Center Hamburg-Eppendorf, Martinistraße 52, 20246 Hamburg, Germany; 2 Department of Neuropathology, University Hospital Magdeburg, Magdeburg, Germany; 3 Department of Neuropathology, University Hospital Heidelberg, Heidelberg, Germany; 4 Institute of Neuropathology, University Medical Center Hamburg-Eppendorf, Martinistraße 52, 20246 Hamburg, Germany; 5 Department of Pediatric Hematology and Oncology, University Medical Center Hamburg-Eppendorf, Martinistraße 52, 20246 Hamburg, Germany; 6 Research Institute Children’s Cancer Center Hamburg, Martinistraße 52, 20251 Hamburg, Germany

Spinal meningiomas account for 1.2–12% of all meningiomas and 25–45% of all spinal tumors. About 20% of intracranial meningiomas and 4.6% of spinal meningiomas recur and require additional treatment. The classification of intracranial meningiomas has evolved considerably in recent years and uses genetic [1, 3, 7] as well as epigenetic parameters [5, 8] in order to more precisely predict the patients’ prognosis and to lay the ground for therapeutic regimens that are adapted to the aggressiveness of a patient’s tumor. Spinal meningiomas are missing in many of the large cohorts that were gathered for molecular profiling of meningiomas and have neither been thoroughly analyzed separately. Therefore, their classification still relies mostly on histopathological findings.

We performed genetic and epigenetic profiling of 65 tumor samples from patients with histologically proven spinal meningioma. Clinical features are described in Supplementary Table 1, online resource, and raw data are accessible at GEO under GSE212449. *T*-distributed Stochastic Neighbor Embedding (*t*-SNE) analysis of genome-wide DNA methylation data shows that most spinal meningiomas separated from cranial meningiomas and formed two distinct clusters (Fig. 1a, b). Sixteen out of 19 cases in cluster 1 (84%) significantly matched the methylation class “benign-2” [8], while cluster 2 was more heterogeneous with only 5/42 samples reaching a significant match (12%, MSC score > 0.9, Fig. 1c and d, Supplementary Fig. 1a–c, online resource). Of 19 meningiomas in cluster 1, 13 (68%) were of the meningothelial subtype, 12 (63%) occurred in the cervical spine, and 13 (68%) occurred in male patients (Chi-square, p < 0.001, Fig. 1d, Supplementary Fig. 1d–i, online resource). Notably, all cases with verified AKT1 E17K mutations resided in cluster 1, thereby making up the majority of this cluster (8/13 cases analyzed for mutations). *NF2* mutations were detected in 24/32 spinal meningiomas (75%) of the second cluster (Supplementary Fig. 1j, online resource), but only in a single tumor in cluster 1. In line with this observation, a hemizygous loss of chromosome 22q was observed in most cluster 2 cases (29/32, 91%), but only in 3/19 cluster 1 cases (16%, *p* < 0.0001, Fig. 1d and e). Other mutations that may occur in meningiomas [6] were not recurrently found in our cohort (Supplementary Table 1, online resource). Seven spinal tumors showed close association with cranial meningioma of intermediate and malignant methylation subclasses according to *t*-SNE analysis (Fig. 1b) and did not show a clear association with cluster 1 or 2 in this context. These cases had a particularly high frequency of cytogenetic aberrations (Fig. 1d and e, marked by asterisks). Collectively, these data suggest that spinal meningiomas encompass two major clinically, genetically, and epigenetically distinct tumor groups, which are either localized primarily in the cervical or the thoracic spine. These data are well in line with recently published work [4] and further complement this work by extensive epigenetic characterization of such tumors. Together, such results may, in the end, lead to a more precise and clinically relevant classification of spinal meningiomas.

**Fig. 1:**
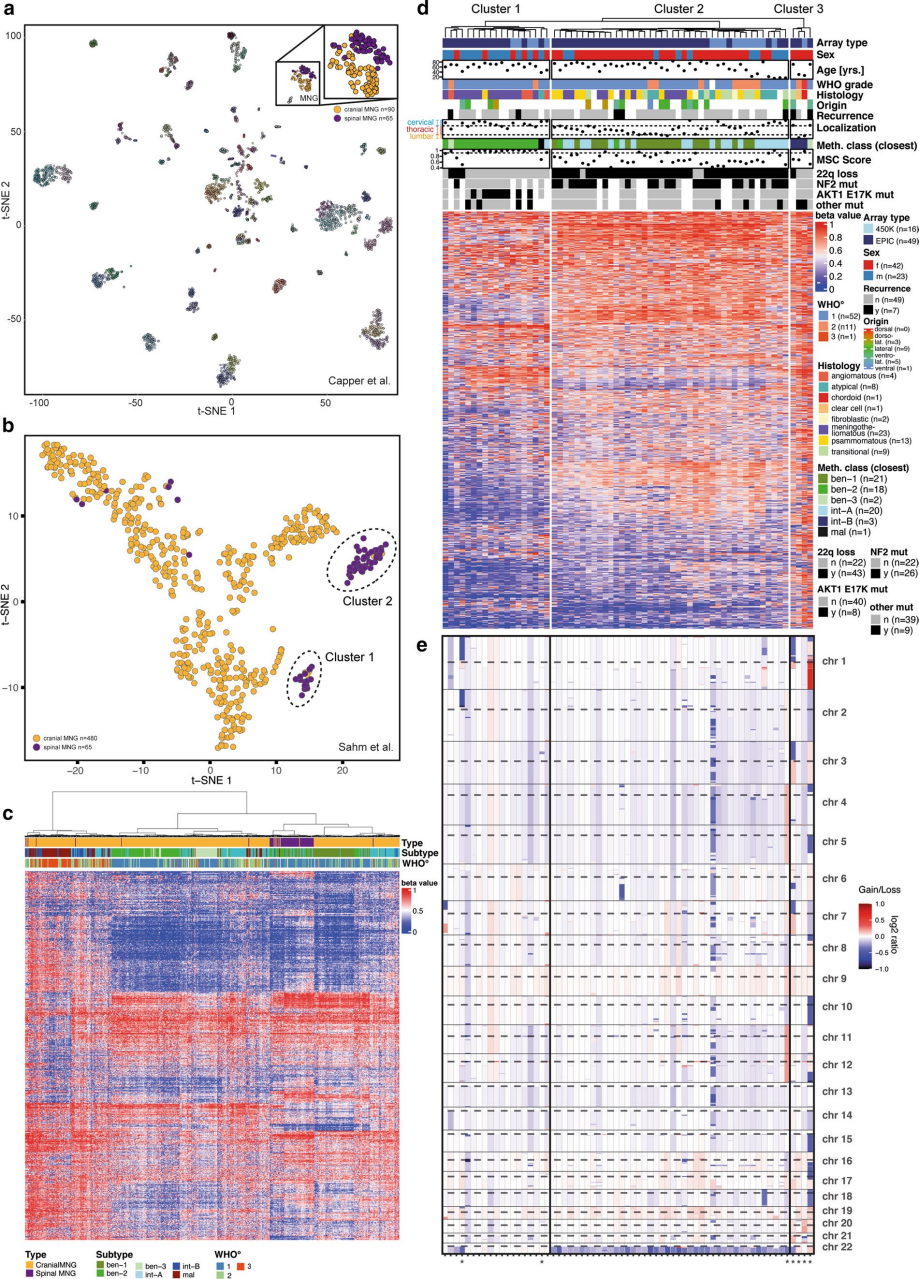
Epigenetic and genetic characterization of spinal meningiomas. A cohort of 65 spinal meningiomas (54 tumors newly acquired, 11 tumors from a reference cohort [8]) was analyzed using DNA methylation arrays and panel-based DNA sequencing. **a** *T*-stochastic neighbor embedding (*T*-SNE) of genome-wide DNA methylation profiles of 65 spinal meningiomas combined with a reference cohort of 2,801 CNS tumors [2] after pairwise Pearson correlation, according to [2]. **b** *T*-SNE analysis of 65 spinal meningiomas combined with a reference cohort of 480 cranial meningiomas [8]. **c** Hierarchical clustering of 10,000 CpG positions with the highest SD from the DNA methylation array datasets used in b (Ward’s linkage method, Euclidean distances). **d** Oncoplot of 65 spinal meningioma with clinical and genetic features. Unsupervised hierarchical clustering of 10,000 CpG positions with highest SD across samples (complete linkage, Euclidean distance). MSC = methylation subclass score, Scores > 0.9 **e** Genome-wide copy number variation profile of spinal meningioma samples from **d** based on DNA methylation arrays. Asterisks mark cases that do not belong to the two main clusters according to b and that mostly reveal a high frequency of cytogenetic aberrations

## Supplementary Information

Below is the link to the electronic supplementary material.

Supplementary file1 (DOCX 13 kb)

Supplementary file2 (TIF 1237 kb)

Supplementary file3 (DOCX 51 kb)
