# Supplementary material for: Genetic and epigenetic profiling identifies two distinct classes of spinal meningiomas
Source: Acta Neuropathol. 2022 Sep 27;144(5):1057–9. doi: 10.1007/s00401-022-02504-6 (PMC9547788; doi:10.1007/s00401-022-02504-6)
Supplement: Supplementary file 1 — Supplementary file1 (DOCX 13 kb) [file 401_2022_2504_MOESM1_ESM.docx]

Supplementary Table 1:

**Clinical characteristics**

Supplementary Figure 1:

**Correlation between clinical parameters and methylation-based subclasses of spinal and cranial meningiomas.** **a - c** tSNE plot after pairwise Pearson correlation from methylation array data of 50 spinal and 480 cranial meningioma samples as seen in Fig.1b. Samples are colored according to their methylation subclass (**a**, Heidelberg meningioma classifier, closest match), their histology **(b)** or by the location at which these tumours occurred **(c)**. Spinal MNGs of Cluster 1 clustered next to ben-2 tumors and mostly retained chromosome 22q, as is characteristic for tumors of this subclass (**a**). Spinal MNGs of Cluster 2 clustered in the vicinity of ben-1 tumours and showed loss of chromosome 22q and/or NF2 mutations, as is characteristic of ben-1 MNGs. Histologically, spinal cluster 1 MNGs show a strong association with meningotheliomatous MNGs (**b**). Spinal cluster 1 MNGs seem to show a closer association with skull base tumours, while spinal cluster 2 meningiomas seem to be molecularly closer related to meningiomas occurring in the posterior fossa (**c**). **d-f** Spinal meningiomas of Clusters 1 and 2 show significant differences in their localization and methylation subclass scores (as calculated by the Heidelberg meningioma classifier) but not in patient age. **g-i** A majority of spinal meningiomas of cluster 1 contain AKT1 E17K mutations, while most spinal cluster 2 tumours show a heterozygous loss of chromosome 22q and/or a NF2 mutation**. j.** lolliplot visualization of NF2 mutation sites.
