# Supplementary figures and images for: Genetic and epigenetic profiling identifies two distinct classes of spinal meningiomas
Source: Acta Neuropathol. 2022 Sep 27;144(5):1057–9. doi: 10.1007/s00401-022-02504-6 (PMC9547788; doi:10.1007/s00401-022-02504-6)

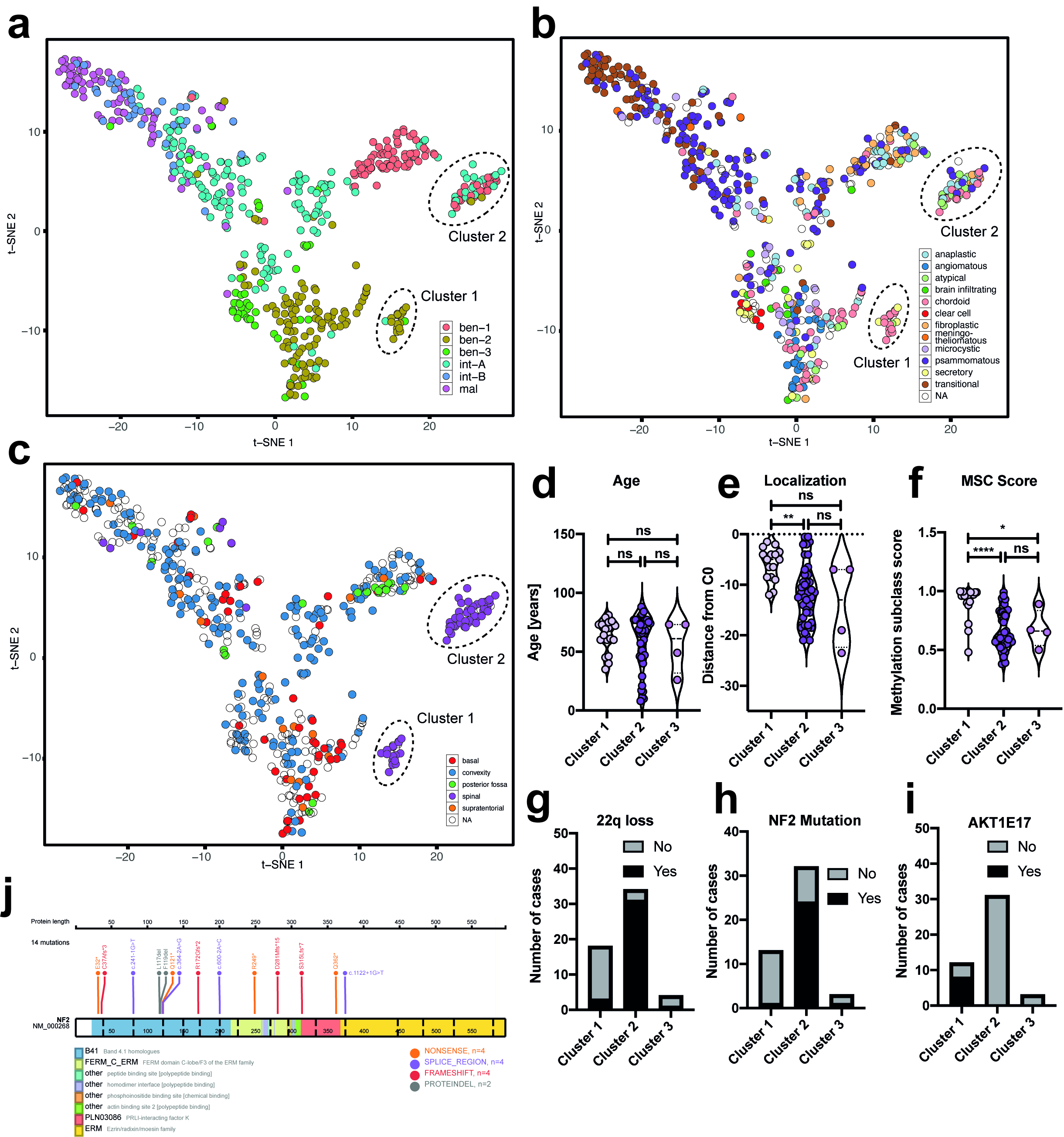

Supplement: Supplementary file 2 — Supplementary file2 (TIF 1237 kb) [file 401_2022_2504_MOESM2_ESM.tif]
