# Supplementary material for: Genetic and epigenetic profiling identifies two distinct classes of spinal meningiomas
Source: Acta Neuropathol. 2022 Sep 27;144(5):1057–9. doi: 10.1007/s00401-022-02504-6 (PMC9547788; doi:10.1007/s00401-022-02504-6)
Supplement: Supplementary file 3 — Supplementary file3 (DOCX 51 kb) [file 401_2022_2504_MOESM3_ESM.docx]

Supplementary Table 1

Clinical, mutational and DNA methylation-based data of the spinal meningioma cohort

| ID | SentrixID | Cluster | Array | Sex | Age | WHO ° | Histology | Position | Resection | Length FUP [months] | Recurrence | Localization | Meth. Subclass | MSC Score | Chr. 22q loss | NF2 mut | AKT1 E17K  mut | TRAF7 mut | PIK3CA mut | PIK3CB mut | PIK3CG mut | POLR2A mut | SMARCE1 mut | SMARCA4 mut |
| --- | --- | --- | --- | --- | --- | --- | --- | --- | --- | --- | --- | --- | --- | --- | --- | --- | --- | --- | --- | --- | --- | --- | --- | --- |
| 1 | 204081740083_R04C01 | 2 | EPIC | m | 77 | 1 | psammomatous | 1.5 | C | 44 | no | C0/C1 | int-A | 0.66 | yes |  |  |  |  |  |  |  |  |  |
| 2 | 204081740083_R05C01 | 1 | EPIC | m | 40 | 1 | meningotheliomatous | 1.5 | C | 5 | no | C4 | ben-2 | 0.48 | yes |  |  |  |  |  |  |  |  |  |
| 3 | 204081740083_R06C01 | 2 | EPIC | f | 43 | 1 | meningotheliomatous | 2 | C | 1 | no | T11 | int-A | 0.62 | yes |  |  |  |  |  |  |  |  |  |
| 4 | 204894630047_R02C01 | 3 | EPIC | f | 73 | 1 | transitional | 1.5 | S | 44 | yes | C7 | int-B | 0.67 | yes |  |  |  |  |  |  |  |  |  |
| 4_2 | 204894630050_R06C01 | 3 | EPIC | f | 73 | 1 |  | 1.5 |  | 44 | yes | C7 | ben-3 | 0.5 | no | no | no | no | no | no | no | no | no | no |
| 5 | 204894630047_R03C01 | 2 | EPIC | f | 65 | 1 | meningotheliomatous | 2 | C | 101 | yes | T7 | int-A | 0.61 | yes | no | no | no | no | no | no | no | no | no |
| 5_2 | 204894630047_R01C01 | 2 | EPIC | f | 65 | 1 |  | 1.5 |  | 101 | yes | T7 | ben-2 | 0.81 | yes |  |  |  |  |  |  |  |  |  |
| 6 | 204894630047_R04C01 | 2 | EPIC | f | 57 | 1 | psammomatous | 2 | C | 2 | no | L1 | ben-1 | 0.59 | yes | no | no | no | no | no | no | no | no | no |
| 7 | 204894630047_R05C01 | 2 | EPIC | f | 60 | 1 | transitional | 2.5 | C | 9 | no | T1 | int-A | 0.48 | yes | yes | no | no | no | no | no | no | no | no |
| 8 | 205107400148_R06C01 | 3 | EPIC | f | 26 | 2 | clear cell |  | C | 104 | no | L4/L5 | int-B | 0.65 | no | no | no | no | no | no | no | no | yes | no |
| 9 | 205107400148_R07C01 | 2 | EPIC | f | 78 | 1 | transitional | 2 | C | 104 | no | T4/T5 | ben-1 | 0.55 | yes | yes | no | no | no | no | no | no | no | no |
| 10 | 205107400148_R08C01 | 2 | EPIC | f | 46 | 1 | meningotheliomatous | 2 | C | 22 | no | L2 | ben-1 | 0.91 | yes | yes | no | no | no | no | no | no | no | no |
| 11 | 205117390143_R05C01 | 2 | EPIC | f | 80 | 2 | atypical | 1 | C | 70 | no | T11/T12 | ben-1 | 0.75 | yes | yes | no | no | no | no | no | no | no | no |
| 12 | 205117390143_R06C01 | 2 | EPIC | m | 10 | 1 | transitional | 2 | C | 123 | yes | C0/C1 | int-A | 0.48 | yes | yes | no | no | no | no | no | no | no | no |
| 13 | 205117390143_R07C01 | 1 | EPIC | m | 73 | 1 | meningotheliomatous | 2.5 | C | 3 | no | C6/C7 | ben-2 | 0.95 | no | no | yes | no | no | no | no | no | no | no |
| 14 | 205117390143_R08C01 | 1 | EPIC | m | 74 | 1 | meningotheliomatous | 2 | C | 18 | no | C5 | ben-2 | 0.96 | no | no | yes | no | no | no | no | no | no | no |
| 15 | 205117390144_R01C01 | 2 | EPIC | m | 77 | 2 | atypical |  | C | 4 | no | T11/T12 | ben-1 | 0.83 | yes | yes | no | no | no | no | no | no | no | no |
| 16 | 205130650118_R05C01 | 2 | EPIC | f | 86 | 2 | atypical |  | C | 0 |  | T4/T5 | ben-1 | 0.5 | no | yes | no | no | no | no | no | no | no | no |
| 17 | 205130650118_R06C01 | 3 | EPIC | f | 49 | 1 | fibroblastic | 2 | C | 78 | no | T12 | ben-1 | 0.89 | no | yes | no | no | no | no | no | no | no | no |
| 18 | 205130650118_R07C01 | 1 | EPIC | m | 60 | 1 | meningotheliomatous | 2 | C | 11 | no | T1/T2 | ben-2 | 0.9 | no | no | no | no | no | no | no | yes | no | no |
| 19 | 205310760050_R01C01 | 1 | EPIC | m | 81 | 1 | meningotheliomatous |  | C | 0 |  | T1/T2 | ben-2 | 0.97 | no | no | yes | no | no | no | no | no | no | no |
| 20 | 205310760050_R02C01 | 2 | EPIC | f | 88 | 1 | meningotheliomatous |  | C | 12 | no | T5/T6 | ben-1 | 0.56 | yes | yes | no | no | no | no | no | no | no | no |
| 21 | 205310760050_R03C01 | 1 | EPIC | f | 70 | 1 | meningotheliomatous |  | C | 0 |  |  | ben-2 | 0.72 | yes | yes | no | no | no | no | no | no | no | no |
| 22 | 205310760050_R04C01 | 2 | EPIC | m | 68 | 1 | meningotheliomatous |  | C | 9 | no | T3/T4 | int-A | 0.57 | yes | no | no | no | no | no | no | no | no | no |
| 23 | 205310760050_R05C01 | 1 | EPIC | m | 61 | 1 | meningotheliomatous |  | C | 0 |  | C3/C4 | ben-2 | 0.88 | no | no | no | no | no | no | no | no | no | no |
| 24 | 205310760050_R06C01 | 2 | EPIC | f | 77 | 1 | meningotheliomatous |  | C | 0 |  | T9/T10 | int-A | 0.57 | yes | no | no | no | no | no | no | no | no | no |
| 25 | 205310760050_R07C01 | 2 | EPIC | m | 66 | 1 | meningotheliomatous |  | C | 0 |  | T3 | ben-1 | 0.63 | yes | no | no | no | no | no | no | no | no | no |
| 26 | 205310760113_R07C01 | 2 | EPIC | f | 74 | 1 | psammomatous |  | C | 0 |  | T11 | ben-1 | 0.55 | yes | yes | no | no | no | no | no | no | no | no |
| 27 | 205310760113_R08C01 | 1 | EPIC | m | 73 | 1 | meningotheliomatous |  | C | 12 | no | C4 | ben-2 | 0.99 | no | no | yes | no | no | no | no | no | no | no |
| 28 | 205437190093_R03C01 | 2 | EPIC | m | 77 | 1 | meningotheliomatous |  | C | 8 | no | T4/T5 | int-A | 0.82 | yes | yes | no | no | no | no | no | no | no | no |
| 29 | 205437190093_R04C01 | 2 | EPIC | f | 82 | 1 | psammomatous |  | C | 0 |  | T5/T6 | ben-1 | 0.84 | yes | yes | no | no | no | no | no | no | no | no |
| 30 | 205437190093_R05C01 | 2 | EPIC | f | 77 | 1 | psammomatous |  | C | 6 | no | T2/T3 | ben-1 | 0.76 | yes | yes | no | no | no | no | no | no | no | no |
| 31 | 205437190093_R06C01 | 1 | EPIC | m | 45 | 1 | meningotheliomatous |  | C | 0 |  | T4/T5 | ben-2 | 0.99 | no | no | yes | no | no | no | no | no | no | no |
| 32 | 205437190093_R07C01 | 2 | EPIC | f | 77 | 1 | psammomatous |  | C | 12 | no | T8/T9 | int-A | 0.53 | no | yes | no | no | no | no | no | no | no | no |
| 33 | 205437190093_R08C01 | 1 | EPIC | m | 60 | 1 | meningotheliomatous |  | C | 0 |  | C1/C2 | ben-2 | 0.97 | no | no | no | yes | yes | no | no | no | no | no |
| 34 | 206144080133_R08C01 | 2 | EPIC | f | 46 | 1 | psammomatous |  |  | 3 | no | T1 | ben-2 | 0.735 | yes | yes | no | no | no | no | no | no | no | no |
| 35 | 206144080139_R07C01 | 2 | EPIC | f | 74 | 1 |  |  |  | 4 | no | T2/T3 | ben-1 | 0.61 | yes | yes | no | no | no | no | no | no | no | no |
| 36 | 206238130054_R03C01 | 1 | EPIC | f | 79 | 1 | psammomatous |  |  | 3 | no | C2/C3 | ben-2 | 0.98 | no | no | yes | no | no | no | no | no | no | no |
| 37 | 206144080133_R06C01 | 2 | EPIC | f | 68 | 1 | meningotheliomatous | 2.5 | C | 4 | no | T5/T6 | ben-1 | 0.94 | yes | yes | no | no | no | no | no | no | no | no |
| 38 | 201465920020_R03C01 | 2 | EPIC | m | 11 | 1 |  |  |  | 0 |  | C4 | int-A | 0.82 | yes | yes | no | yes | no | no | no | no | no | no |
| 39 | 202093110119_R02C01 | 1 | EPIC | m | 72 | 2 | angiomatous |  |  |  | yes | T5 | ben-3 | 0.96 | yes |  |  |  |  |  |  |  |  |  |
| 40 | 202229250094_R07C01 | 1 | EPIC | m | 35 | 1 | atypical |  |  | 0 |  | C1-C3 | mal | 0.66 | no | no | no | no | no | no | no | no | no | no |
| 41 | 202284800135_R04C01 | 2 | EPIC | f | 16 | 1 | atypical |  | S | 0 |  | C4 | int-A | 0.89 | yes | no | no | no | no | no | no | no | no | no |
| 42 | 206359150112_R06C01 | 2 | EPIC | f | 57 | 1 | transitional |  |  | 3 |  | T11/T12 | ben-1 | 0.80 | yes |  |  |  |  |  |  |  |  |  |
| 43 | 206100100135_R01C01 | 1 | EPIC | f | 60 | 1 | angiomatous |  |  | 5 |  | C5 | ben-2 | 0.96 | no |  |  |  |  |  |  |  |  |  |
| 44 | 206359150112_R04C01 | 2 | EPIC | f | 73 | 1 | psammomatous |  |  | 2 |  | T12/L1 | ben-1 | 0.93 | yes |  |  |  |  |  |  |  |  |  |
| 45 | 205772290008_R01C01 | 2 | EPIC | f | 40 | 1 | meningotheliomatous |  |  | 8 |  | T8 | ben-1 | 0.79 | yes |  |  |  |  |  |  |  |  |  |
| 46 | 205689110150_R07C01 | 2 | EPIC | f | 74 | 1 | psammomatous |  |  | 11 |  | T6 | int-A | 0.38 | yes |  |  |  |  |  |  |  |  |  |
| 47 | 205292900127_R05C01 | 2 | EPIC | f | 51 | 1 | transitional |  |  | 13 |  | T2/T3 | int-A | 0.61 | yes |  |  |  |  |  |  |  |  |  |
| 48 | 10006823123_R01C02 | 2 | 450K | f | 47 | 2 | transitional |  | C | 1 | no | T10/T11 | ben-1 | 0.52 | yes | no | no | no | no | no | yes | no | no | no |
| 49 | 10006823123_R05C02 | 2 | 450K | f | 75 | 1 | transitional |  | C | 3 | no | C6/C7 | int-A | 0.42 | yes |  |  |  |  |  |  |  |  |  |
| 50 | 200394870072_R01C01 | 2 | 450K | m | 17 | 2 | transitional |  |  | 0 |  | T11 | ben-1 | 0.59 | yes | yes | no | no | no | no | no | no | no | no |
| 51 | 3998523016_R03C01 | 2 | 450K | f |  |  | atypical |  |  | 140 | no |  | int-A | 0.39 | yes | yes | no | no | no | no | no | no | no | no |
| 52 | 3999834036_R01C01 | 2 | 450K | f | 69 | 1 | psammomatous |  |  | 61 | no | C2 | ben-1 | 0.44 | yes | yes | no | no | no | no | yes | no | no | no |
| 53 | 3999834036_R01C02 | 2 | 450K | f | 73 | 2 | psammomatous |  | C | 46 | no | T3 | int-A | 0.59 | yes | yes | no | no | no | no | no | no | no | no |
| 54 | 3999834036_R06C01 | 2 | 450K | m | 29 | 2 | psammomatous |  | S | 67 | no | T8/T9 | int-A | 0.65 | yes |  |  |  |  |  |  |  |  |  |
| 55 | 3999834045_R01C02 | 1 | 450K | f | 69 | 1 | meningotheliomatous |  |  | 143 | no | C4 | ben-2 | 0.997 | no |  |  |  |  |  |  |  |  |  |
| 56 | 3999834045_R04C01 | 1 | 450K | f | 64 | 2 | meningotheliomatous |  |  | 1 | no | T2 | ben-2 | 0.998 | no |  |  |  |  |  |  |  |  |  |
| 57 | 3999834071_R03C01 | 1 | 450K | m | 75 | 1 | meningotheliomatous |  |  |  | yes | C | ben-2 | 0.99 | no | no | yes | no | no | no | no | no | no | yes |
| 58 | 9376561057_R03C01 | 2 | 450K | f | 84 | 1 | atypical |  |  | 16 | no | C7 | int-A | 0.57 | yes | yes | no | no | no | no | no | no | no | no |
| 59 | 9533774014_R06C02 | 2 | 450K | m | 8 | 1 | atypical |  |  | 0 |  | C2 | int-A | 0.99 | yes | yes | no | no | no | no | no | no | no | no |
| 60 | 9611519054_R05C01 | 2 | 450K | f | 21 | 3 | chordoid |  |  | 0 |  | L2 | int-B | 0.96 | no | no | no | no | no | no | no | no | yes | no |
| 61 | 9741950149_R05C01 | 2 | 450K | f | 45 | 2 | fibroblastic |  |  | 0 |  | T4 | ben-1 | 0.60 | yes | yes | no | no | no | no | no | no | no | no |
| 62 | 9878827220_R01C02 | 1 | 450K | m | 76 | 1 | angiomatous |  |  | 0 |  | C6/C7 | ben-2 | 0.99 | no | no | yes | no | no | yes | no | no | no | no |
| 63 | 9878827220_R06C01 | 1 | 450K | f | 46 | 1 | angiomatous |  | C | 7 | no | C1/C2 | int-A | 0.91 | no |  |  |  |  |  |  |  |  |  |

**Position:** 1 = ventral; 2 = lateral; 3 = dorsal

**Localization:** Number of vertebrae starting from skull base, on which the tumor was located

**Resection:** Extent of surgical resection. C = complete; S = subtotal

**Methylation Subclass and MSC Score** as obtained by the Heidelberg meningioma methylation classifier (v.2.4, www.molecularneuropathology.org)

Missing data is indicated by empty cells.
